# 2-Amino-*N*-(4-methyl­phenyl­sulfon­yl)-*N*-phenyl­benzene­sulfonamide

**DOI:** 10.1107/S1600536808007447

**Published:** 2008-03-29

**Authors:** Zu-Wei Song

**Affiliations:** a College of Science, Qingdao Agricultural University, Qingdao 266109, People’s Republic of China

## Abstract

In the title mol­ecule, C_19_H_18_N_2_O_4_S_2_, the phenyl ring makes dihedral angles of 33.99 (2) and 43.70 (3)° with the two methyl-substituted benzene rings. Inter­molecular N—H⋯O hydrogen bonds link the mol­ecules into centrosymmetric dimers. The crystal packing exhibits weak inter­molecular C—H⋯O hydrogen bonds.

## Related literature

For the crystal structures of related compounds, see: Henschel *et al.* (1996 ▶). For details of the biological activities of sulfon­amide-containing compounds, see: Kamoshita *et al.* (1987 ▶). For related literature, see: Allen *et al.* (1987 ▶); Zhang *et al.* (2007 ▶).
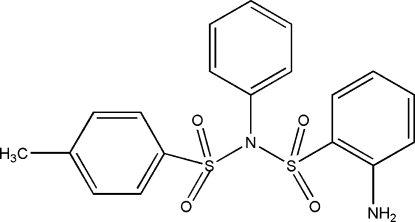


## Experimental

### 

#### Crystal data

C_19_H_18_N_2_O_4_S_2_
*M* *_r_* = 402.47
Monoclinic, 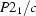

*a* = 14.6245 (3) Å
*b* = 10.0454 (2) Å
*c* = 13.4735 (4) Å
β = 107.478 (2)°
*V* = 1887.99 (7) Å^3^
*Z* = 4
Mo *K*α radiation
μ = 0.31 mm^−1^
*T* = 293 (2) K
0.52 × 0.32 × 0.25 mm

#### Data collection

Rigaku R-AXIS RAPID IP area-detector diffractometer
Absorption correction: multi-scan (*ABSCOR*; Higashi 1995 ▶) *T*_min_ = 0.855, *T*_max_ = 0.926
17437 measured reflections
4312 independent reflections
3531 reflections with *I* > 2σ(*I*)
*R*_int_ = 0.038

#### Refinement

*R*[*F* ^2^ > 2σ(*F* ^2^)] = 0.039
*wR*(*F* ^2^) = 0.121
*S* = 1.07
4312 reflections
245 parameters
H-atom parameters constrained
Δρ_max_ = 0.40 e Å^−3^
Δρ_min_ = −0.26 e Å^−3^

### 

Data collection: *RAPID-AUTO* (Rigaku, 2004 ▶); cell refinement: *RAPID-AUTO*; data reduction: *RAPID-AUTO*; program(s) used to solve structure: *SHELXTL* (Sheldrick, 2008 ▶); program(s) used to refine structure: *SHELXTL*; molecular graphics: *SHELXTL*; software used to prepare material for publication: *SHELXTL*.

## Supplementary Material

Crystal structure: contains datablocks I, global. DOI: 10.1107/S1600536808007447/cv2388sup1.cif

Structure factors: contains datablocks I. DOI: 10.1107/S1600536808007447/cv2388Isup2.hkl

Additional supplementary materials:  crystallographic information; 3D view; checkCIF report

## Figures and Tables

**Table 1:** Hydrogen-bond geometry (Å, °)

*D*—H⋯*A*	*D*—H	H⋯*A*	*D*⋯*A*	*D*—H⋯*A*
N2—H2*B*⋯O3^i^	0.86	2.20	3.062 (2)	176
C19—H19*A*⋯O1^ii^	0.96	2.57	3.517 (3)	169
C19—H19*C*⋯O4^iii^	0.96	2.58	3.538 (3)	174
N2—H2*C*⋯O2	0.86	2.23	2.893 (2)	133

Symmetry codes: (i) 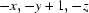; (ii) 
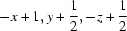
; (iii) 
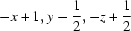
.

**Fig. 1.**
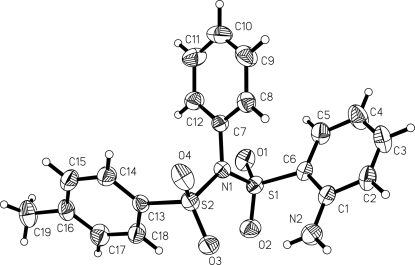
The molecular structure of (I) showing the atomic numbering and 40% probability displacement ellipsoids.

## supplementary crystallographic information

### Comment

Many compounds containing sulfonimide groups possess a broad spectrum of biological activities and can be used as herbicides (Kamoshita *et al.*, 1987). In addition, some compounds containing sulfonimide groups can be used as catalysts (Zhang *et al.*, 2007). Herein we report the crystal structure of the title compound, (I).

In (I) (Fig. 1), all bond lengths and angles are normal (Allen *et al.*, 1987) and in a good agreement with those reported previously (Henschel *et al.*, 1996). The phenyl ring (C7—C12) makes the dihedral angles of 33.99 (2) and 43.70 (3)°, respectively, with two benzene rings (C1—C6/N1 and C13—C19). The intermolecular N—H···O hydrogen bonds (Table 1) link the molecules into centrosymmetric dimers. The crystal packing exhibits also weak intermolecular C—H···O hydrogen bonds (Table 1).

### Experimental

A solution of methylsulfonyl chloride (1 mmol) was dissolved in anhydrous CH_2_Cl_2_ (10 ml), and dropwise added over a period of 10 min to a solution of 2-amino-*N*-methyl-benzenesulfonamide (1 mmol) and DMAP_2_ (3 mmol) in CH_2_Cl_2_ (10 ml) at 273 K. The mixture was stirred for 4 h at room temperature. The organic phase was washed with 2 N HCl twice, and dried over anhydrous Na_2_SO_4_. The solvent was removed and the residue was purified by flash chromatography (1:1 cyclohexane:dichloromethane) to give (I) as a white solid (294 mg, 73%). Single crystals suitable for X-ray measurements were obtained by recrystallization from ethanol and dichloromethane at room temperature.

### Refinement

H atoms were positioned geometrically with C—H = 0.93 or 0.96 Å, N—H=0.86 Å, and refined using a riding model, with *U*_iso_(H) = 1.2 (1.5 for methyl groups) times *U*_eq_(C, N).

### Crystal data

**Table:**

C_19_H_18_N_2_O_4_S_2_	*F*(000) = 840
*M**_r_* = 402.47	*D*_x_ = 1.416 Mg m^−^^3^
Monoclinic, *P*2_1_/*c*	Mo *K*α radiation, λ = 0.71073 Å
Hall symbol: -P 2ybc	Cell parameters from 2652 reflections
*a* = 14.6245 (3) Å	θ = 2.6–25.6°
*b* = 10.0454 (2) Å	µ = 0.31 mm^−^^1^
*c* = 13.4735 (4) Å	*T* = 293 K
β = 107.478 (2)°	Platelet, yellow
*V* = 1887.99 (7) Å^3^	0.52 × 0.32 × 0.25 mm
*Z* = 4	

### Data collection

**Table:**

Rigaku R-AXIS RAPID IP area-detector diffractometer	4312 independent reflections
Radiation source: Rotating Anode	3531 reflections with *I* > 2σ(*I*)
graphite	*R*_int_ = 0.038
ω oscillation scans	θ_max_ = 27.5°, θ_min_ = 1.5°
Absorption correction: multi-scan (*ABSCOR*; Higashi 1995)	*h* = −18→18
*T*_min_ = 0.855, *T*_max_ = 0.927	*k* = −13→13
17437 measured reflections	*l* = −17→17

### Refinement

**Table:**

Refinement on *F*^2^	Primary atom site location: structure-invariant direct methods
Least-squares matrix: full	Secondary atom site location: difference Fourier map
*R*[*F*^2^ > 2σ(*F*^2^)] = 0.039	Hydrogen site location: inferred from neighbouring sites
*wR*(*F*^2^) = 0.121	H-atom parameters constrained
*S* = 1.07	*w* = 1/[σ^2^(*F*_o_^2^) + (0.0647*P*)^2^ + 0.3731*P*] where *P* = (*F*_o_^2^ + 2*F*_c_^2^)/3
4312 reflections	(Δ/σ)_max_ = 0.001
245 parameters	Δρ_max_ = 0.40 e Å^−^^3^
0 restraints	Δρ_min_ = −0.26 e Å^−^^3^

### Special details

**Table:**

Geometry. All e.s.d.'s (except the e.s.d. in the dihedral angle between two l.s. planes) are estimated using the full covariance matrix. The cell e.s.d.'s are taken into account individually in the estimation of e.s.d.'s in distances, angles and torsion angles; correlations between e.s.d.'s in cell parameters are only used when they are defined by crystal symmetry. An approximate (isotropic) treatment of cell e.s.d.'s is used for estimating e.s.d.'s involving l.s. planes.
Refinement. Refinement of *F*^2^ against ALL reflections. The weighted *R*-factor *wR* and goodness of fit *S* are based on *F*^2^, conventional *R*-factors *R* are based on *F*, with *F* set to zero for negative *F*^2^. The threshold expression of *F*^2^ > σ(*F*^2^) is used only for calculating *R*-factors(gt) *etc*. and is not relevant to the choice of reflections for refinement. *R*-factors based on *F*^2^ are statistically about twice as large as those based on *F*, and *R*- factors based on ALL data will be even larger.

### Fractional atomic coordinates and isotropic or equivalent isotropic displacement parameters (Å^2^)

**Table:**

	*x*	*y*	*z*	*U*_iso_*/*U*_eq_	
S1	0.18008 (3)	0.28081 (5)	0.24068 (4)	0.04085 (14)	
S2	0.24948 (3)	0.51762 (5)	0.14469 (3)	0.04136 (14)	
O1	0.24248 (10)	0.20876 (14)	0.32523 (12)	0.0550 (4)	
O2	0.16312 (11)	0.23184 (16)	0.13781 (11)	0.0573 (4)	
O3	0.18520 (10)	0.46901 (19)	0.05005 (10)	0.0595 (4)	
O4	0.24789 (12)	0.65454 (14)	0.17142 (12)	0.0571 (4)	
N1	0.22958 (11)	0.43445 (16)	0.24350 (11)	0.0386 (3)	
N2	−0.00565 (13)	0.3985 (2)	0.09409 (13)	0.0651 (6)	
H2B	−0.0542	0.4371	0.0515	0.078*	
H2C	0.0415	0.3727	0.0731	0.078*	
C1	−0.00348 (13)	0.3782 (2)	0.19378 (15)	0.0431 (4)	
C2	−0.08053 (14)	0.4204 (2)	0.22702 (18)	0.0541 (5)	
H2D	−0.1332	0.4599	0.1794	0.065*	
C3	−0.08033 (17)	0.4052 (3)	0.3272 (2)	0.0667 (6)	
H3B	−0.1326	0.4346	0.3469	0.080*	
C4	−0.00295 (18)	0.3463 (3)	0.4010 (2)	0.0691 (7)	
H4B	−0.0034	0.3370	0.4696	0.083*	
C5	0.07352 (16)	0.3022 (2)	0.37162 (16)	0.0540 (5)	
H5A	0.1251	0.2618	0.4202	0.065*	
C6	0.07447 (12)	0.31772 (19)	0.26886 (14)	0.0399 (4)	
C7	0.25885 (13)	0.49745 (18)	0.34475 (13)	0.0375 (4)	
C8	0.19747 (14)	0.5849 (2)	0.37156 (16)	0.0459 (4)	
H8A	0.1393	0.6076	0.3234	0.055*	
C9	0.22318 (18)	0.6384 (2)	0.47042 (19)	0.0586 (6)	
H9A	0.1821	0.6970	0.4894	0.070*	
C10	0.3098 (2)	0.6048 (3)	0.54093 (18)	0.0650 (6)	
H10A	0.3268	0.6406	0.6077	0.078*	
C11	0.37175 (19)	0.5185 (3)	0.51344 (18)	0.0640 (6)	
H11A	0.4302	0.4969	0.5615	0.077*	
C12	0.34685 (14)	0.4643 (2)	0.41479 (15)	0.0497 (5)	
H12A	0.3883	0.4066	0.3956	0.060*	
C13	0.36661 (12)	0.47731 (19)	0.14622 (13)	0.0384 (4)	
C14	0.44113 (15)	0.5618 (2)	0.19508 (16)	0.0514 (5)	
H14A	0.4301	0.6370	0.2301	0.062*	
C15	0.53243 (15)	0.5326 (2)	0.19103 (18)	0.0562 (5)	
H15A	0.5829	0.5890	0.2239	0.067*	
C16	0.55032 (14)	0.4216 (2)	0.13933 (16)	0.0494 (5)	
C17	0.47460 (17)	0.3389 (2)	0.09216 (19)	0.0590 (6)	
H17A	0.4860	0.2635	0.0576	0.071*	
C18	0.38235 (15)	0.3642 (2)	0.09434 (18)	0.0523 (5)	
H18A	0.3322	0.3071	0.0620	0.063*	
C19	0.64941 (17)	0.3926 (3)	0.1334 (2)	0.0718 (7)	
H19A	0.6850	0.4742	0.1402	0.108*	
H19B	0.6451	0.3521	0.0676	0.108*	
H19C	0.6815	0.3331	0.1887	0.108*	

### Atomic displacement parameters (Å^2^)

**Table:**

	*U*^11^	*U*^22^	*U*^33^	*U*^12^	*U*^13^	*U*^23^
S1	0.0370 (2)	0.0369 (2)	0.0459 (3)	0.00456 (18)	0.00835 (18)	−0.00020 (18)
S2	0.0365 (2)	0.0506 (3)	0.0373 (2)	0.00913 (19)	0.01168 (18)	0.00992 (19)
O1	0.0466 (8)	0.0449 (8)	0.0664 (9)	0.0112 (6)	0.0064 (7)	0.0118 (7)
O2	0.0573 (9)	0.0564 (9)	0.0567 (8)	0.0020 (7)	0.0148 (7)	−0.0172 (7)
O3	0.0401 (7)	0.0969 (12)	0.0360 (7)	0.0060 (8)	0.0031 (6)	0.0088 (7)
O4	0.0668 (10)	0.0444 (8)	0.0697 (9)	0.0160 (7)	0.0354 (8)	0.0182 (7)
N1	0.0390 (8)	0.0422 (8)	0.0361 (7)	−0.0013 (6)	0.0133 (6)	0.0008 (6)
N2	0.0426 (9)	0.1027 (17)	0.0438 (9)	0.0165 (10)	0.0037 (7)	0.0140 (10)
C1	0.0340 (8)	0.0449 (10)	0.0468 (10)	−0.0028 (8)	0.0067 (7)	0.0042 (8)
C2	0.0347 (9)	0.0589 (13)	0.0679 (13)	0.0040 (9)	0.0145 (9)	0.0136 (11)
C3	0.0513 (12)	0.0765 (16)	0.0834 (16)	0.0059 (12)	0.0371 (12)	0.0146 (14)
C4	0.0654 (15)	0.0889 (19)	0.0633 (14)	0.0073 (14)	0.0350 (12)	0.0226 (13)
C5	0.0492 (11)	0.0615 (13)	0.0510 (11)	0.0031 (10)	0.0147 (9)	0.0201 (10)
C6	0.0326 (8)	0.0389 (9)	0.0460 (9)	−0.0015 (7)	0.0085 (7)	0.0053 (8)
C7	0.0395 (9)	0.0388 (9)	0.0352 (8)	−0.0038 (7)	0.0128 (7)	−0.0001 (7)
C8	0.0444 (10)	0.0430 (10)	0.0532 (11)	0.0009 (8)	0.0189 (8)	−0.0003 (8)
C9	0.0690 (14)	0.0522 (12)	0.0646 (13)	−0.0004 (11)	0.0353 (11)	−0.0124 (10)
C10	0.0861 (18)	0.0632 (14)	0.0464 (12)	−0.0049 (13)	0.0208 (11)	−0.0143 (10)
C11	0.0643 (14)	0.0713 (15)	0.0459 (11)	0.0031 (12)	0.0005 (10)	−0.0055 (11)
C12	0.0429 (10)	0.0574 (12)	0.0459 (10)	0.0052 (9)	0.0089 (8)	−0.0034 (9)
C13	0.0341 (8)	0.0462 (10)	0.0355 (8)	0.0042 (7)	0.0116 (7)	0.0048 (7)
C14	0.0454 (11)	0.0551 (12)	0.0541 (11)	−0.0023 (9)	0.0157 (9)	−0.0107 (10)
C15	0.0390 (10)	0.0646 (14)	0.0620 (13)	−0.0083 (10)	0.0104 (9)	−0.0056 (11)
C16	0.0384 (10)	0.0553 (12)	0.0573 (12)	0.0063 (9)	0.0189 (9)	0.0121 (9)
C17	0.0546 (12)	0.0540 (12)	0.0748 (15)	0.0035 (10)	0.0291 (11)	−0.0118 (11)
C18	0.0416 (10)	0.0533 (12)	0.0627 (12)	−0.0037 (9)	0.0168 (9)	−0.0106 (10)
C19	0.0484 (12)	0.0763 (17)	0.100 (2)	0.0100 (12)	0.0359 (13)	0.0127 (15)

### Geometric parameters (Å, °)

**Table:**

S1—O2	1.4206 (15)	C8—C9	1.379 (3)
S1—O1	1.4248 (14)	C8—H8A	0.9300
S1—N1	1.7002 (16)	C9—C10	1.378 (3)
S1—C6	1.7378 (19)	C9—H9A	0.9300
S2—O4	1.4237 (16)	C10—C11	1.382 (4)
S2—O3	1.4241 (15)	C10—H10A	0.9300
S2—N1	1.6698 (15)	C11—C12	1.380 (3)
S2—C13	1.7543 (18)	C11—H11A	0.9300
N1—C7	1.447 (2)	C12—H12A	0.9300
N2—C1	1.349 (3)	C13—C14	1.382 (3)
N2—H2B	0.8600	C13—C18	1.389 (3)
N2—H2C	0.8600	C14—C15	1.384 (3)
C1—C2	1.398 (3)	C14—H14A	0.9300
C1—C6	1.414 (2)	C15—C16	1.380 (3)
C2—C3	1.357 (3)	C15—H15A	0.9300
C2—H2D	0.9300	C16—C17	1.377 (3)
C3—C4	1.394 (3)	C16—C19	1.504 (3)
C3—H3B	0.9300	C17—C18	1.382 (3)
C4—C5	1.367 (3)	C17—H17A	0.9300
C4—H4B	0.9300	C18—H18A	0.9300
C5—C6	1.397 (3)	C19—H19A	0.9600
C5—H5A	0.9300	C19—H19B	0.9600
C7—C8	1.380 (3)	C19—H19C	0.9600
C7—C12	1.389 (3)		
			
O2—S1—O1	119.11 (10)	C9—C8—C7	119.4 (2)
O2—S1—N1	106.54 (9)	C9—C8—H8A	120.3
O1—S1—N1	106.09 (8)	C7—C8—H8A	120.3
O2—S1—C6	112.39 (9)	C10—C9—C8	119.9 (2)
O1—S1—C6	109.36 (9)	C10—C9—H9A	120.1
N1—S1—C6	101.64 (8)	C8—C9—H9A	120.1
O4—S2—O3	120.08 (10)	C9—C10—C11	120.6 (2)
O4—S2—N1	105.17 (8)	C9—C10—H10A	119.7
O3—S2—N1	108.43 (9)	C11—C10—H10A	119.7
O4—S2—C13	108.07 (9)	C12—C11—C10	120.1 (2)
O3—S2—C13	108.12 (9)	C12—C11—H11A	120.0
N1—S2—C13	106.17 (8)	C10—C11—H11A	120.0
C7—N1—S2	117.37 (12)	C11—C12—C7	118.8 (2)
C7—N1—S1	114.93 (11)	C11—C12—H12A	120.6
S2—N1—S1	127.69 (9)	C7—C12—H12A	120.6
C1—N2—H2B	120.0	C14—C13—C18	121.05 (18)
C1—N2—H2C	120.0	C14—C13—S2	119.43 (15)
H2B—N2—H2C	120.0	C18—C13—S2	119.45 (15)
N2—C1—C2	119.54 (18)	C13—C14—C15	118.9 (2)
N2—C1—C6	123.36 (18)	C13—C14—H14A	120.6
C2—C1—C6	117.10 (18)	C15—C14—H14A	120.6
C3—C2—C1	121.5 (2)	C16—C15—C14	121.5 (2)
C3—C2—H2D	119.2	C16—C15—H15A	119.3
C1—C2—H2D	119.2	C14—C15—H15A	119.3
C2—C3—C4	121.1 (2)	C17—C16—C15	118.16 (19)
C2—C3—H3B	119.5	C17—C16—C19	120.9 (2)
C4—C3—H3B	119.5	C15—C16—C19	121.0 (2)
C5—C4—C3	119.3 (2)	C16—C17—C18	122.3 (2)
C5—C4—H4B	120.3	C16—C17—H17A	118.9
C3—C4—H4B	120.3	C18—C17—H17A	118.9
C4—C5—C6	120.2 (2)	C17—C18—C13	118.12 (19)
C4—C5—H5A	119.9	C17—C18—H18A	120.9
C6—C5—H5A	119.9	C13—C18—H18A	120.9
C5—C6—C1	120.73 (18)	C16—C19—H19A	109.5
C5—C6—S1	117.82 (14)	C16—C19—H19B	109.5
C1—C6—S1	120.92 (14)	H19A—C19—H19B	109.5
C8—C7—C12	121.20 (18)	C16—C19—H19C	109.5
C8—C7—N1	119.59 (16)	H19A—C19—H19C	109.5
C12—C7—N1	119.15 (17)	H19B—C19—H19C	109.5
			
O4—S2—N1—C7	−27.73 (15)	S2—N1—C7—C8	85.99 (19)
O3—S2—N1—C7	−157.36 (13)	S1—N1—C7—C8	−95.07 (18)
C13—S2—N1—C7	86.67 (14)	S2—N1—C7—C12	−96.67 (19)
O4—S2—N1—S1	153.48 (12)	S1—N1—C7—C12	82.28 (19)
O3—S2—N1—S1	23.85 (15)	C12—C7—C8—C9	−1.3 (3)
C13—S2—N1—S1	−92.12 (13)	N1—C7—C8—C9	176.02 (18)
O2—S1—N1—C7	−175.64 (13)	C7—C8—C9—C10	0.4 (3)
O1—S1—N1—C7	−47.78 (15)	C8—C9—C10—C11	0.4 (4)
C6—S1—N1—C7	66.53 (14)	C9—C10—C11—C12	−0.4 (4)
O2—S1—N1—S2	3.18 (14)	C10—C11—C12—C7	−0.4 (4)
O1—S1—N1—S2	131.04 (12)	C8—C7—C12—C11	1.2 (3)
C6—S1—N1—S2	−114.65 (12)	N1—C7—C12—C11	−176.1 (2)
N2—C1—C2—C3	−178.3 (2)	O4—S2—C13—C14	16.10 (18)
C6—C1—C2—C3	0.6 (3)	O3—S2—C13—C14	147.52 (16)
C1—C2—C3—C4	−0.2 (4)	N1—S2—C13—C14	−96.30 (17)
C2—C3—C4—C5	−0.6 (4)	O4—S2—C13—C18	−161.03 (16)
C3—C4—C5—C6	0.8 (4)	O3—S2—C13—C18	−29.62 (19)
C4—C5—C6—C1	−0.4 (3)	N1—S2—C13—C18	86.56 (17)
C4—C5—C6—S1	171.3 (2)	C18—C13—C14—C15	0.5 (3)
N2—C1—C6—C5	178.5 (2)	S2—C13—C14—C15	−176.61 (17)
C2—C1—C6—C5	−0.3 (3)	C13—C14—C15—C16	0.1 (3)
N2—C1—C6—S1	7.1 (3)	C14—C15—C16—C17	−0.6 (3)
C2—C1—C6—S1	−171.74 (16)	C14—C15—C16—C19	178.6 (2)
O2—S1—C6—C5	148.79 (17)	C15—C16—C17—C18	0.6 (4)
O1—S1—C6—C5	14.18 (19)	C19—C16—C17—C18	−178.7 (2)
N1—S1—C6—C5	−97.68 (17)	C16—C17—C18—C13	0.0 (4)
O2—S1—C6—C1	−39.51 (19)	C14—C13—C18—C17	−0.5 (3)
O1—S1—C6—C1	−174.13 (15)	S2—C13—C18—C17	176.55 (17)
N1—S1—C6—C1	74.01 (17)		

### Hydrogen-bond geometry (Å, °)

**Table:**

*D*—H···*A*	*D*—H	H···*A*	*D*···*A*	*D*—H···*A*
N2—H2B···O3^i^	0.86	2.20	3.062 (2)	176.
C19—H19A···O1^ii^	0.96	2.57	3.517 (3)	169.
C19—H19C···O4^iii^	0.96	2.58	3.538 (3)	174.
N2—H2C···O2	0.86	2.23	2.893 (2)	133.

Symmetry codes: (i) −*x*, −*y*+1, −*z*; (ii) −*x*+1, *y*+1/2, −*z*+1/2; (iii) −*x*+1, *y*−1/2, −*z*+1/2.
